# Bacterial Diversity Associated with Wild Caught *Anopheles* Mosquitoes from Dak Nong Province, Vietnam Using Culture and DNA Fingerprint

**DOI:** 10.1371/journal.pone.0118634

**Published:** 2015-03-06

**Authors:** Chung Thuy Ngo, Fabien Aujoulat, Francisco Veas, Estelle Jumas-Bilak, Sylvie Manguin

**Affiliations:** 1 Institut de Recherche pour le Développement (IRD), UMR-MD3, Faculté de Pharmacie, Montpellier, France; 2 National Institute of Veterinary Research, Hanoi, Vietnam; 3 University Montpellier 1, UMR 5119 ECOSYM, Equipe Pathogènes et Environnements, Faculté de Pharmacie, Montpellier, France; Centro de Pesquisas René Rachou, BRAZIL

## Abstract

**Background:**

Microbiota of *Anopheles* midgut can modulate vector immunity and block *Plasmodium* development. Investigation on the bacterial biodiversity in *Anopheles*, and specifically on the identification of bacteria that might be used in malaria transmission blocking approaches, has been mainly conducted on malaria vectors of Africa. Vietnam is an endemic country for both malaria and Bancroftian filariasis whose parasitic agents can be transmitted by the same *Anopheles* species. No information on the microbiota of *Anopheles* mosquitoes in Vietnam was available previous to this study.

**Method:**

The culture dependent approach, using different mediums, and culture independent (16S rRNA PCR – TTGE) method were used to investigate the bacterial biodiversity in the abdomen of 5 *Anopheles* species collected from Dak Nong Province, central-south Vietnam. Molecular methods, sequencing and phylogenetic analysis were used to characterize the microbiota.

**Results and Discussion:**

The microbiota in wild-caught *Anopheles* was diverse with the presence of 47 bacterial OTUs belonging to 30 genera, including bacterial genera impacting *Plasmodium* development. The bacteria were affiliated with 4 phyla, *Actinobacteria*, *Bacteroidetes*, *Firmicutes* and *Proteobacteria*, the latter being the dominant phylum. Four bacterial genera are newly described in *Anopheles* mosquitoes including *Coxiella*, *Yersinia*, *Xanthomonas*, and *Knoellia*. The bacterial diversity per specimen was low ranging from 1 to 4. The results show the importance of pairing culture and fingerprint methods to better screen the bacterial community in *Anopheles* mosquitoes.

**Conclusion:**

Sampled *Anopheles* species from central-south Vietnam contained a diverse bacterial microbiota that needs to be investigated further in order to develop new malaria control approaches. The combination of both culture and DNA fingerprint methods allowed a thorough and complementary screening of the bacterial community in *Anopheles* mosquitoes.

## Introduction

*Anopheles* mosquitoes can be vectors of human pathogens responsible of infectious diseases such as malaria and lymphatic filariasis, which represent a great public health challenge in many tropical countries. In Vietnam, malaria remains the most important vector-borne parasitic disease with a higher prevalence in forested regions, in particular along the international borders with Cambodia. The goal of the National Malaria Control Program (NMCP) is to eliminate malaria by 2020 of the 63 provinces in the country. Presently 40 provinces have no local malaria transmission, 15 are in elimination phase and 8 in pre-elimination phase including those with hyper-endemic malaria foci [1].

*Plasmodium falciparum*, the parasite responsible for the majority of recorded malaria (63%) in Vietnam, followed by *P*. *vivax* (37%) may share the same *Anopheles* vector species with *Wuchereria bancrofti*, the nematode responsible for Bancroftian lymphatic filariasis (BLF), for which only limited information is available [2]. Many of these *Anopheles* vectors belong to sibling species complexes or taxonomic groups of closely related species with different degrees of involvement in the transmission of parasites. The inherent difficulties to differentiate these species morphologically [3,4] creates operational problems in providing targeted vector control for controlling the pathogens they carry [1].

The complex factors allowing the development of a pathogen to reach the infective stage in a mosquito are incompletely known. On the 539 described species of mosquitoes within the *Anopheles* genus [5], only 60 to 70 are capable of transmitting malaria and BLF [6,7]. In vector-parasite interactions, the mosquito gut represents the first point of contact between parasites ingested and the vector’s epithelial surfaces. In the midgut, where the parasites begin their life cycle, the tens of thousands of *Plasmodium* gametocytes that might be ingested by a mosquito, less than five oocysts might be produced [8]. The factors responsible for this drastic reduction are still poorly understood. Recent studies showed that one of these factors concerns the primordial role played by bacteria naturally present in the mosquito midgut [9–13]. There is a growing interest on bacterial biodiversity in *Anopheles* mosquitoes and particularly those based on the identification of bacteria that might be used for malaria transmission blocking based on bacterial genetic changes to deliver anti-parasite molecules or a paratransgenic approach to control [13–20].

Recent studies have been conducted to investigate bacterial species in field-collected *Anopheles* mosquitoes using culture-dependent and/or culture-independent approaches focusing on primary vector species only [14,15,21–23]. To date, no study has been conducted on natural bacteria diversity in *Anopheles* mosquitoes from Vietnam combining these two methodologies.

The objective of our study was to characterize bacteria in the abdomen of wild-caught *Anopheles* species collected in Dak Nong Province, Vietnam, using both culture-dependent and culture-independent (DNA fingerprint) methods.

## Materials and Methods

### Ethical statement

The specimens used in this study were provided by the Military Preventive Medicine Centre, Ho Chi Minh City (Vietnam) who organized the field study and obtained all necessary permits. The Vietnam People’s Army Department of Military Medicine approved the study. Mosquito collections were done with the approval of the head of each village and the owner and occupants of the houses where mosquitoes were collected. Mosquito collectors gave their consent and were diagnosed and treated free-of-charge in the event of a malaria episode during the study in accordance with the national drug policy of Vietnam.

### Samples

Specimens belonging to 5 *Anopheles* species, including *Anopheles barbumbrosus*, *An*. *crawfordi*, *An*. *dirus*, *An*. *maculatus* and *An*. *gigas*, were collected from 6 sites located in Dak Ngo Commune, Tuy Duc District, Dak Nong Province, Vietnam (11°59’N 107°42’E—central Highlands). These *Anopheles* specimens were collected between November and December 2010 during 10 consecutive nights using several methods, including mechanical light traps, human-landing catches, cow-baited captures and resting collections [24].

Initial *Anopheles* mosquito identification was morphologically done in the field by sorting out each taxon. Specimens that belonged to the Dirus Complex or the Maculatus Group were individually identified to species level using the appropriate PCR-based method as described by Walton et al. [25,26]. Each individual was split in two paired sections, head-thorax for species identification and abdomen for bacteria analysis, and stored at -80°C until analyzed. One hundred abdomens of wild-caught females were used for the bacterial study.

### Bacterial culture and DNA extraction

*Anopheles* abdomens were surface rinsed twice in sterilized DNA-free water, and each abdomen was thoroughly disrupted using a sterilized tissue crusher device in 150 μl of sterilized DNA-free water. Then, 10 μl of this suspension was spread on each prepared culture medium plate: blood sheep agar, R2A and *Acetobacter* agar. The inoculated agar plates were incubated at 30°C during 72 hrs and checked every 24 hrs for bacterial growth. Colonial morphotypes were differentiated and subcultured on a new agar plate and incubated at the primary plates to obtain pure isolates. The bacterial isolates were transferred to tryptic soy agar plate and incubated at 30°C during 24 hrs. Then, isolated colonies were suspended in purified DNA-free water until turbidity equal to McFarland N°5 (about 1.5 10^9^ bacteria/mL) was reached, boiled for 10 min and frozen at -20°C for raw DNA extraction. Each bacterial isolate were stored at -80°C in tryptic soy broth with 15% glycerol. Whole DNA was extracted from 100 μl of mosquito abdomen suspension using the Master Pure Gram Positive DNA purification kit as recommended by the supplier (Epicentre Biotechnologics, Madison, USA). The purified and raw DNAs were kept at -20°C before further analyses.

### PCR

For PCR-TTGE experiments, the V2–V3 region of the 16S rRNA gene of bacteria was amplified using the primers HDA1/HDA2 [27]; HDA1: 5’-ACTC CTA CGG GAG GCA GCA GT-3’, HDA2: 5’-GTA TTA CCG CGG CTG CTG GCA-3’. A 40-bp clamp, named GC (5’-CGC CCG GGG CGC GCC CCG GGC GGG GCG GGG GCA CGG GGG G-3’) flanked the 5’ extremity of HDA1 [28] in order to form HDA1-GC. PCR was performed using an Eppendorf thermal cycler (Eppendorf, Le Pecq, France) and 0.5 ml tubes. The reaction mixture (50 μl) contained 2.5 units of Taq DNA Polymerase (FastStart High Fidelity PCR system, Roche, Meylan, France), 0.2 mM of dNTPs, 0.2 mM of each primer and 1 μl of abdomen content DNA in the appropriate reaction buffer. Thermal cycling conditions consisted of an initial denaturation step at 95°C for 2 min, then 35 cycles each consisting of denaturation at 95°C for 1 min, annealing at 62°C for 30 s and extention at 72°C for 1 min, with a final extension at 72°C for 7 min. A nearly complete 16S rRNA gene sequence was amplified with DNA from the bacterial isolates as template using the universal primers 27f [29] and 1492r, as described [30]. PCR amplifications were checked by DNA electrophoresis in 1.5% agarose gels containing ethidium bromide and visualized under ultraviolet light.

For the *Anopheles* specimens that did not show presence of bacteria, an ITS2-PCR was processed in order to verify that absence of microbiota detection was not due to failed DNA extraction. The protocol used a reaction mixture (25 μl) containing 5 μl of 5X PCR reaction buffer, 1.5 mM of MgCl_2_, 0.5 units of Tfi DNA polymerase, 0.2 mM of dNTPs, 0.2 μM of each universal primer, ITS2A (5’-TGT GAA CTG CAG GAC ACA T-3’) and ITS2B (5’-TAT GCT TAA ATT CAG GGG GT-3’) and 3 μl of abdomen content DNA in the appropriate reaction buffer. Thermal cycling conditions consisted of an initial denaturation step at 94°C for 2 min, then 40 cycles each consisting of denaturation at 94°C for 30 s, annealing at 51°C for 30 s and extention at 72°C for 1 min, with a final extension at 72°C for 10 min.

### Temporal Temperature Gel Electrophoresis

Temporal Temperature Gel Electrophoresis (TTGE) was performed using the DCode universal mutation detection system (Bio-Rad Laboratories, Marne-la-Coquette, France) in gels that were 16 cm × 16 cm by 1 mm. The gels (60 ml) were composed of 8% (wt/vol) acrylamide-bisacrylamide (37.5:1), 7 M urea, 60 ml of N,N,N’,N’-tetramethylethylenediamine (TEMED), and 0.1% (wt/vol) ammonium persulfate. Gels were run with 1X Tris–acetate–EDTA buffer at pH 8.4. A volume of 5 μl of DNA was loaded on gel with 5 μl of in-house dye marker (saccharose 50%, Bromophenol Blue 0.1%) using capillary tips. Denaturing electrophoresis was performed at 46 V with a temperature ramp from 63°C to 70°C during 16 hrs (0.4°C/h), after a pre-migration of 15 min at 20 V. Gels were stained with ethidium bromide solution (5μg/ml) for 20 min, washed with de-ionized water, viewed using a UV trans-illumination system (Vilbert-Lourmat, France) and photographed.

### TTGE band sequencing and OTU affiliation

TTGE bands were excised and the DNA was eluted with 50 μl of elution buffer (EB) of the Qiaquick PCR purification kit (Qiagen, Courtabeuf, France) overnight at 37°C before PCR amplification with HDA1/HDA2 used without GC clamp. The reaction conditions were identical to those described above. PCR products were sequenced on an ABI 3730xl sequencer (Cogenics, Meylan, France). Each sequencing chromatograph was visually inspected and corrected. The sequences were analyzed by comparison with Genbank (http://www.ncbi.nlm.nih.gov/) and Ribosomal Databases Project 2 (RDPII) (http://rdp.cme.msu.edu/) using Basic Local Alignment Search Tool (BLAST) and Seqmatch programs, respectively. The sequence with the highest percentage was used for OTU affiliation. A sequence was affiliated to a species-level OTU when the percent of sequence similarity with the species type strain was above 99.0% [31]. This value is over the recognized cut-off value for the delineation of species [32], but warrants high stringency for species-level OTU affiliation. Below 99.0%, the sequence is affiliated to the genus of the reference sequence with the highest percentage. When several species reference sequences match equally, affiliation was done to the genus level or to a group of species, if relevant. For example, sequence with 99.5% in similarity to both *Aeromonas caviae* and *Aeromonas hydrophila* was only assigned to the genus *Aeromonas*. The same rule was applied for the taxonomic level higher than the genus level. On each TTGE gel, about 50% of the bands were sequenced, the others being affiliated to an OTU by comparison of their migration distance with that of sequenced bands. The species richness was estimated by the determination of the crude Diversity Index (DI), corresponding to the number of different OTUs identified from each mosquito. Rarefaction analysis was carried out using the online program *Analytic Rarefaction* available at http://strata.uga.edu/software/Software.html.

### Phylogeny

Sequences obtained herein and sequences selected from RDPII (http://rdp.cme.msu.edu) were used for phylogenetic analysis. Sequences were aligned using the ClustalW program. Maximum-likelihood (ML) analysis was performed using phylogenetic analysis at http://www.phylogeny.fr [33]. The general time-reversible (GTR) model plus gamma distribution and invariant sites was used as the best substitution model determinated by Akaike criteria (Modeltest v3.7 software) [34]. ML bootstrap support was computed after 100 reinterations. The sequence of *Chlamydia trachomatis* HAR-13^T^ (NR_025888) was used as outgroup sequence in order to place an artificial tree root.

## Results and Discussion

### Taxonomic diversity of bacteria in the abdomen of Anopheles adults caught in Dak Nong, Vietnam

From 100 *Anopheles* specimens belonging to 5 species, 83% showed the presence of bacteria in either PCR-TTGE or culture. Bacteria were not detected in 17 specimens (6 *An*. *maculatus*, 4 *An*. *barbumbrosus*, 3 *An*. *dirus*, 2 *An*. *crawfordi*, and 2 *An*. *gigas*). For these, an ITS2-PCR assay was performed showing that the absence of microbiota detection was not due to failed DNA extraction, but likely because of low bacterial inoculum under the threshold of detection by techniques used. The percentages of samples per *Anopheles* species that could not be analyzed for bacteria diversity were 21.4%, 30.8%, 13.0%, 16.2%, and 8.3% respectively. Bacteria diversity was analyzed in all positive samples. Detection of bacteria in 83% of tested samples lies between 15% of *Anopheles* mosquitoes from Kenya reported by Lindh et al. [15] and those reported by Boissière et al. [35] in which 100% of *Anopheles gambiae* in Cameroon were found with midgut bacteria using a pyrosequencing method.

The bacterial microbiota of 100 *Anopheles* abdomens, characterized by DNA fingerprinting and culture methods, found 47 bacterial OTUs belonging to 30 genera (Table 1) within 17 families in the phyla *Actinobacteria*, *Bacteroidetes*, *Firmicutes*, and *Proteobacteria*. The diversity detected in this study was likely underestimated as suggested by the rarefaction curve presented in supplementary data. Twenty out of 30 genera belonged to *Proteobacteria*, which were present in 73% of the specimens tested (Table 1). This result is in accordance with those recently reported by Rani et al. [22], Djadid et al. [20] and Boissière et al. [35], who concluded that *Proteobacteria* was the dominant phylum in bacterial communities found in *An*. *stephensi* collected in India, *An*. *stephensi* and *An*. *maculipennis* from Iran, and *An*. *gambiae* from Cameroon, respectively. Moreover, the four bacterial phyla reported herein, have also been identified in *Anopheles* mosquitoes from Kenya [15], Iran [14,20], *An*. *gambiae* in Cameroon [35], and in *Aedes aegypti*, a vector of various viral pathogens [36], suggesting that at least a fraction of microbiota is common to different mosquito species and genera.

**Table 1:** Bacterial genera and OTUs in abdomens of *Anopheles* species collected in Vietnam.

Phyla	Genera, family or order / OTUs belonged to genera	*An*. *Barbumbrosus* (= 13)	*An*. *crawfordi* (= 12)	*An*. *dirus* (= 23)	*An*. *gigas* (= 24)	*An*. *maculatus* (= 28)	Total
***Proteobacteria***	*Acetobacteraceae*	*** ***	*** ***	*** ***	*** ***	2	2
	*Acinetobacter* */*Acinetobacter*, *A*. *junii*, *Acinetobacter* sp.	3	7	11	19	13	53
	Asaia **/*A*. *spathodeae*		1				1
	Bartonella **/ *Bartonella* sp.				1		1
	*Coxiella* (1) /*Diplorickettsia massiliensis*			1			1
	*Cellvibrio* /*C*. *ostraviensis*			1			1
	*Enhydrobacter* /*Enhydrobacter*, *E*. *aerosaccus*, *Enhydrobacter* sp.	1	2			1	4
	*Enterobacter*/*E*. *aerogenes*			2			2
	*Hafnia* /*Hafnia paralvei*			1			1
	*Klebsiella* /*Klebsiella pneumoniae*		1				1
	*Serratia / Serratia* sp.		2				2
	*Tatumella* /*Tatumella* sp.			1			1
	*Thorsellia* /*Thorsellia anophelis*		1		3		4
	*Yersinia* (1) /*Yersinia* sp.		2				2
	*Moraxella* /*Moraxella osloensis*			1			1
	*Novosphingobium* / *Novosphingobium* sp.			3		13	16
	*Pseudomonas* /*P*. *aeruginosa*, *Pseudomonas* sp.		2	1			3
	*Sphingomonadaceae*			7	6	2	15
	*Sphingomonadales*			6		7	13
	*Sphingobium* /*Sphingobium* spp.			1			1
	*Sphingomonas* /*Sphingomonas* sp.				1		1
	*Stenotrophomonas* /*S*. *maltophilia*, *Stenotrophomonas* sp.			2			2
	Xanthomonas ** (1) /*Xanthomonas* sp.	1					1
***Firmicutes***	*Staphylococcus* */*S*. *pasteuri*, *S*. *sciuri*, *S*. *warneri*	1	1		2	1	5
	Bacillus **/*Bacillus* sp.				1		1
	*Enterococcus* /*Enterococcus faecium*		1				1
***Actinobacteria***	Brachybacterium **/ *Brachybacterium* spp.	1	1				2
	Brevibacterium **/*Brevibacterium* sp.		1				1
	Janibacter **/*Janibacter* sp.					1	1
	Leucobacter **/*Leucobacter chromiiresistens*	1					1
	*Microbacterium* */*Microbacterium* sp., *M*. *radiodurans*, *M*. *testaceum*	2					2
	*Micrococcaceae*		1				1
***Bacteroidetes***	*Chryseobacterium* /*Chryseobacterium* sp.	1					1
	Flavobacteriaceae **	2					2
	Knoellia ** (1) /*Knoellia* sp.	1					1
**Total**		**14**	**23**	**38**	**33**	**40**	**148**
**Diversity index**		**1.1**	**1.9**	**1.7**	**1.4**	**1.4**	**1.5**

* Genera or OTU revealed by both culture dependent and independent methods.

** Genera or OTU revealed only in culture pathway. (1) Genera or OTU newly identified in *Anopheles* mosquitoes according to the review by Manguin et al. [37] and the present study. Number of samples colonized for each genus or family/OTU per *Anopheles* species is shown in the case box.

Within the *Proteobacteria*, the genus *Acinetobacter* was dominant and present in 53% of the samples, followed by *Novosphingobium* at 16% (Table 1). *Acinetobacter* was also the most common bacteria identified in *An*. *gambiae* collected in Cameroon [35], as well as other *Anopheles* species collected from Iran, India, Kenya and Mali [14,22,38]. The genus *Novosphingobium* (Family *Sphingomonadaceae*) contents numerous bacteria species known to be metabolically versatile and occupy different ecological niches [39–41]. *Novosphingobium* is a genus recently reported in *Anopheles* mosquitoes [42–45] and being the second most commonly encountered genus in the *Anopheles* from Vietnam, further study will be needed to investigate its association with the mosquito and host pathogens. Other members of *Sphingomonadaceae* accounted for 15% of our samples and included genera such as *Sphingobium* and *Sphingomonas*. This latter genus has also been detected in *An*. *gambiae* [18,35].

Beside these more prevalent genera, intermediate occurrence was observed for *Staphylococcus* (5%), *Enhydrobacter* and *Thorsellia* (4%), and *Pseudomonas* (3%). *Thorsellia anophelis* was found in 4 specimens belonging to *An*. *crawfordi* (n = 1) and *An*. *gigas* (n = 3) (Table 1). This bacteria was first isolated and described in 2006 as a new species from *An*. *arabiensis* [46], and has also been reported as the dominant bacterium in *An*. *gambiae* adults from Kenya [47]. Six genera (*Brachybacterium*, *Enterobacter*, *Microbacterium*, *Serratia*, *Stenotrophomonas*, and *Yersinia*) displayed a lower prevalence rate with a presence in 2 individuals each, whereas the remaining 18 genera were detected in only one specimen (1%) from samples tested (Table 1). Among these minority genera, *Chryseobacterium* detected in *An*. *barbumbrosus*, has been reported in *Anopheles* from Kenya [23], larvae and adults from Iran [14], and from other aquatic animals (fish) and various habitats [48,49]. In fact, the latter studies indicated a direct link between the composition of gut microbiota in adult mosquitoes and the bacterial richness of the native aquatic source from which the hosts were derived [12,50].

Bacteria belonging to the family *Enterobacteriaceae* were identified from 10 specimens in 3 *Anopheles* species, *An*. *crawfordi*, *An*. *dirus*, and *An*. *gigas* (Table 1), belonging to seven genera including *Enterobacter*, *Hafnia*, *Tatumella*, *Thorsellia*, *Serratia*, *Yersinia*, and *Klebsiella*. Within *Enterobacteriaceae* positive samples, one specimen of *An*. *crawfordi* was colonized with three enterobacterial genera, *Serratia*, *Yersinia* and *Klebsiella* (data not shown). The genera *Enterobacter* [9,12,50] and *Serratia* [10,15,22,51] have been regarded as having a role in the development cycle of *Plasmodium* in *Anopheles*. *Klebsiella* has also been isolated in the midgut of *An*. *gambiae* collected from Kenya and Mali [38].

Four bacterial genera have been newly detected such as *Coxiella*, *Yersinia*, *Xanthomonas*, and *Knoellia* (Table 1), not yet reported in *Anopheles* mosquitoes either from our previous work by Manguin et al. [37], or recent articles and reviews [42–45,52]. These results suggest that the bacterial diversity associated with *Anopheles* remains underestimated and that some individuals of *Anopheles* populations from Dak Nong, Vietnam displayed important and interesting microbiota diversity. However, the number of different OTUs per specimen (range from 1 to 4) and the correspondant diversity index (DI) (range from 1.1 to 1.9) were relatively low (Table 1). *Anopheles crawfordi* microbiota displayed the highest bacterial diversity (DI = 1.9) but the diversity among the different *Anopheles* species did not differ significantly (p<0.05, Kruskal Wallis test) (Table 1). Rani et al. [22] observing midgut bacterial diversity of lab-reared and field-collected *An*. *stephensi* (both larvae and adults) from India, reported 53 bacterial genera from the midgut with biodiversity index values ranging from 2.75 to 3.49 for field-collected mosquitoes. Therefore, the biodiversity of microbiota in *Anopheles* mosquitoes is influenced by the environment where the mosquito was collected as demonstrated by Boissiere et al [35] with *An*. *gambiae* collected from different areas in Cameroon.

### Comparison of cultivable and molecular microbiota diversity

Among the 100 specimens studied, 52 *Anopheles* were analyzed using both culture and 16S rRNA genes PCR-TTGE fingerprinting. The panel of culture media was chosen accordingly to the diversity previously described for *Anopheles* microbiota [15,35,37]. Only 13 samples (25%) produced positive cultures. No positive sample was found for the 3 *An*. *dirus* specimens. TTGE fingerprinting appeared a more suitable method for bacteria detection in the *Anopheles* abdomen as distinct TTGE patterns were observed in 26/52 (50%) samples. Culture-dependent and culture-independent methods gave congruent results in 38.5% of paired samples (4 positive and 17 negative samples). Twenty-two positive samples by PCR-TTGE were bacteria negative by culture whereas 9 positive samples by culture were undetectable by PCR-TTGE (data not shown).

**Fig 1:**
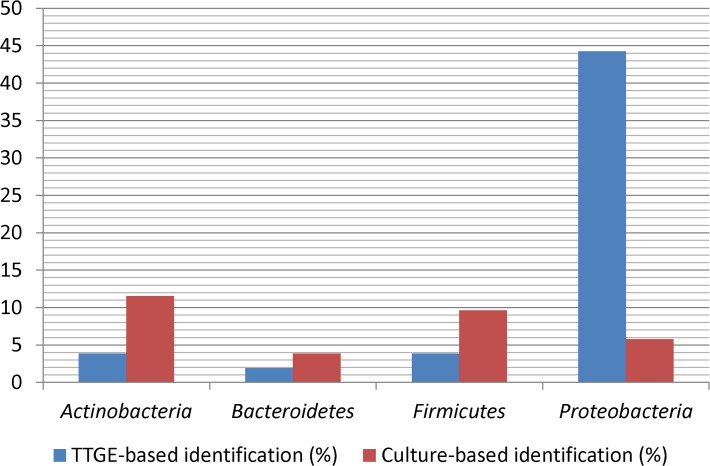
Prevalence of identified isolates or sequences in bacterial phyla for either TTGE- or culture-based method. Values showed the percentage of positive samples within 52 *Anopheles* specimens.

**Fig 2:**
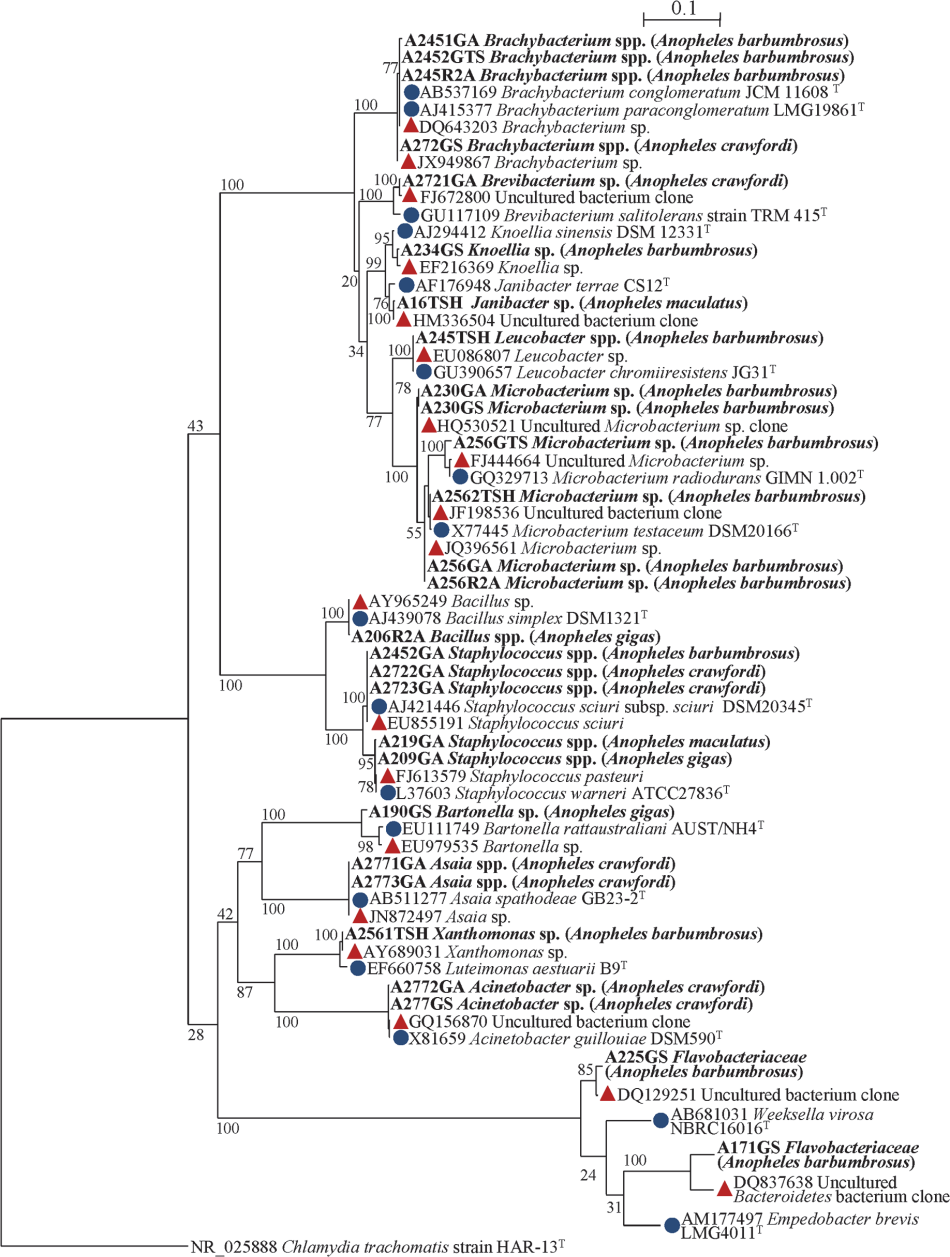
Maximum-likelihood phylogenetic tree showing the position of bacterial strains culture-isolated from abdomens of *Anopheles* species collected in Dak Nong, Vietnam. The horizontal lines show genetic distance. The numbers at the nodes are support values estimated with 100 bootstrap replicates. The scale bar indicates the number of substitutions per nucleotide position. *Chlamydia trachomatis* HAR-13^T^ was used as the outgroup bacteria. The sequences of isolates are shown in bold, the closest sequences (red triangle) and the sequences of the closest species type strain (blue circle) are shown with their GenBank accession number and their annotation. More information on the sequences used is detailed in Table 2.

A total of 28 bacterial strains were isolated and subjected to identification by 16S rRNA gene sequencing. The size of the sequences ranged between 900 and 1,000 bp allowing species-level affiliation in majority of cases. Affiliations into taxonomic levels (species, genus, family, and phylum) are given in Table 2. *Microbacterium* (Phylum *Actinobacteria*) present in 6 specimens was the dominant genus of cultivable bacteria in *Anopheles* sample abdomens, followed by *Staphylococcus* (Phylum *Firmicutes*) (5 isolates) and *Brachybacterium* (Phylum *Actinobacteria*) (4 isolates) (Table 2). These 3 predominant cultivable genera belong to Gram-positive bacterial phyla, mainly *Actinobacteria* (Fig. 1). A 16S rRNA gene sequences-based phylogeny was reconstructed. The ML tree in Fig. 2 showed the phylo-taxonomic position of the bacteria isolated in *Anopheles*. Several isolates were mostly related to uncultured clones and few of them were related to newly described bacterial species [53–57] (Fig. 2).

**Table 2:** Sequence analysis of bacterial isolates obtained from the abdomens of 5 wild-caught *Anopheles* species by culture-dependent method.

N°	Isolate	Sequence size (bp)	Identification	Phylum	Family	Nearest type species RDPII Species name/GenBank accession number/Identity	Closest relative RDPIIGenBank accession number/Identity	*Anopheles* species	GenBank accession number
1	A190GS	1026	*Bartonella* sp.	*Proteobacteria*	*Bartonellaceae*	*Bartonella rattaustraliani*/EU111749/0.855	EU979535/0.862	*An*. *gigas*	KP027793
2	A171GS	1014	*Flavobacteriaceae*	*Bacteroidetes*	*Flavobacteriaceae*	*Empedobacter brevis*/AM177497/0.568	DQ837638/0.746	*An*. *barbumbrosus*	KP027794
3	A225GS	991	*Flavobacteriaceae*	*Bacteroidetes*	*Flavobacteriaceae*	*Wautersiella falsenii*/AM084341/0.624 *Weeksella virosa*/AB681031	DQ129251/0.931	*An*. *barbumbrosus*	KP027795
4	A230GA	978	*Microbacterium* sp.	*Actinobacteria*	*Microbacteriaceae*	*Microbacterium testaceum*/ X77445/0.910	HQ530521/0.965	*An*. *barbumbrosus*	KP027796
5	A230GS	982	*Microbacterium* sp.	*Actinobacteria*	*Microbacteriaceae*	*Microbacterium testaceum/* X77445/0.909	GQ250443/0.954	*An*. *barbumbrosus*	KP027797
6	A234GS	992	*Knoellia* sp.	*Actinobacteria*	*Intrasporangiaceae*	*Knoellia sinensis*/AJ294412/0.921	EF216369/0.937	*An*. *barbumbrosus*	KP027798
7	A2721GA	1001	*Brevibacterium* sp.	*Actinobacteria*	*Brevibacteriaceae*	*Brevibacterium salitolerans*/ *halotolerans*/GU117109/0.866	FJ672800/0.979	*An*. *crawfordi*	KP027799
8	A2722GA	1017	*Staphylococcus sciuri*	*Firmicutes*	*Staphylococcaceae*	*Staphylococcus sciuri*/AJ421446/0.987	AB188210/0.987	*An*. *crawfordi*	KP027800
9	A2723GA	1017	*Staphylococcus sciuri*	*Firmicutes*	*Staphylococcaceae*	*Staphylococcus sciuri*/AJ421446/0.990	EU419917/0.990	*An*. *crawfordi*	KP027801
10	A272GS	994	*Brachybacterium* spp.	*Actinobacteria*	*Dermabacteraceae*	*Brachybacterium paraconglomeratum*/ *conglomeratum*/AJ415377/AB537169/0.960	JX949867/0.978	*An*. *crawfordi*	KP027802
11	A2771GA	1027	*Asaia spathodeae*	*Proteobacteria*	*Acetobacteraceae*	*Asaia spathodeae*/AB511277/0.990	JN872497/0.990	*An*. *crawfordi*	KP027803
12	A2772GA	990	*Acinetobacter* sp.	*Proteobacteria*	*Moraxellaceae*	*Acinetobacter guillouiae*/X81659/0.969	GQ156870/0.976	*An*. *crawfordi*	KP027804
13	A2773GA	1023	*Asaia spathodeae*	*Proteobacteria*	*Acetobacteraceae*	*Asaia spathodeae*/AB511277/0.996	JX445138/0.996	*An*. *crawfordi*	KP027805
14	A277GS	992	*Acinetobacter* sp.	*Proteobacteria*	*Moraxellaceae*	*Acinetobacter guillouiae*/X81659/0.971	GQ156853/0.983	*An*. *crawfordi*	KP027806
15	A219GA	1023	*Staphylococcus warneri*	*Firmicutes*	*Staphylococcaceae*	*Staphylococcus warneri*/L37603/0.986	FM872679/0.992	*An*. *maculatus*	KP027807
16	A16TSH	999	*Janibacter* sp.	*Actinobacteria*	*Intrasporangiaceae*	*Janibacter terrae*/AF176948/0.898	HM336504/0.960	*An*. *maculatus*	KP027808
17	A206R2A	1014	*Bacillus* sp.	*Firmicutes*	*Bacillaceae*	*Bacillus simplex*/AJ439078/0.962	AY965249/0.971	*An*. *gigas*	KP027809
18	A209GA	994	*Staphylococcus warneri*	*Firmicutes*	*Staphylococcaceae*	*Staphylococcus warneri*/L37603/0.987	FJ613579/0.994	*An*. *gigas*	KP027810
19	A2561TSH	1012	*Xanthomonas* sp.	*Proteobacteria*	*Xanthomonadaceae*	*Luteimonas aestuarii*/EF660758/0.899	AY689031/0.995	*An*. *barbumbrosus*	KP027811
20	A2562TSH	1002	*Microbacterium* sp.	*Actinobacteria*	*Microbacteriaceae*	*Microbacterium testaceum*/ X77445/0.963	JF198536/0.990	*An*. *barbumbrosus*	KP027812
21	A256GA	989	*Microbacterium* sp.	*Actinobacteria*	*Microbacteriaceae*	*Microbacterium testaceum*/ X77445/0.945	JQ396561/0.985	*An*. *barbumbrosus*	KP027813
22	A256GTS	994	*Microbacterium* sp.	*Actinobacteria*	*Microbacteriaceae*	*Microbacterium radiodurans*/ GQ329713/0.915	FJ444664/0.930	*An*. *barbumbrosus*	KP027814
23	A256R2A	990	*Microbacterium* sp.	*Actinobacteria*	*Microbacteriaceae*	*Microbacterium testaceum*/ X77445/0.943	JQ396561/0.985	*An*. *barbumbrosus*	KP027815
24	A2451GA	988	*Brachybacterium* spp.	*Actinobacteria*	*Dermabacteraceae*	*Brachybacterium conglomeratum*/ *paraconglomeratum*/AB537169/AJ415377/0.951	DQ643203/0.951	*An*. *barbumbrosus*	KP027816
25	A2452GA	991	*Staphylococcus sciuri*	*Firmicutes*	*Staphylococcaceae*	*Staphylococcus sciuri*/AJ421446/0.984	EU855191/0.985	*An*. *barbumbrosus*	KP027817
26	A245R2A	991	*Brachybacterium* spp.	*Actinobacteria*	*Dermabacteraceae*	*Brachybacterium paraconglomeratum*/ *conglomeratum*/AJ415377/AB537169/0.977	EU086801/0.977	*An*. *barbumbrosus*	KP027818
27	A245TSH	992	*Leucobacter chromiiresistens*	*Actinobacteria*	*Microbacteriaceae*	*Leucobacter chromiiresistens*/GU390657/0.983	EU086807/0.989	*An*. *barbumbrosus*	KP027819
28	A2452GTS	998	*Brachybacterium* spp.	*Actinobacteria*	*Dermabacteraceae*	*Brachybacterium paraconglomeratum*/ *conglomeratum*/AJ415377/AB537169/0.977	JF274910/0.989	*An*. *barbumbrosus*	KP027820

**Fig 3:**
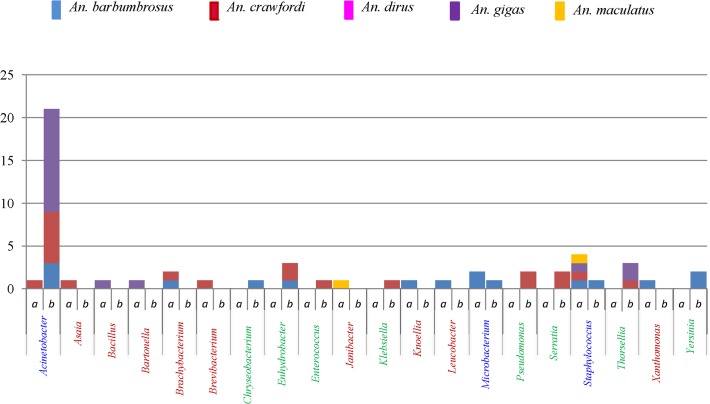
Bacterial genera detected in 52 wild-caught *Anopheles* mosquitoes using of culture-dependent (a) and culture-independent (b) methods, displayed by the number of positive samples in each *Anopheles* species. Out of 3 *An*. *dirus* analyzed, no specimen showed detectable bacteria. Number of genera detected per method such as 9 by culture-dependent (red characters), 8 by culture-independent (green characters) and 3 genera detected by both methods (blue characters).

On the same 52 samples, 39 sequences obtained from TTGE bands showed bacteria classified into 13 OTUs of 11 genera belonging to 8 bacterial families. Out of 11 bacterial genera detected by the culture-independent method (Fig. 3), 7 (8 OTUs) belonged to *Proteobacteria*, a Gram-negative phylum that was also the dominant bacterial phylum detected by PCR-TTGE (Fig. 1).

Among the 20 bacterial genera detected from the 52 samples on which both culture and fingerprint methods were applied, there were only 3 common detected genera (*Acinetobacter*, *Microbacterium*, *Staphylococcus*), 9 and 8 genera were detected by either culture-dependent or culture-independent methods, respectively (Fig. 3), showing the importance of combining these two methods for increasing the detection efficiency of greater microbiota biodiversity.

Discordance between culture-dependent and-independent methods is in accordance with Lindh et al. [15] who showed that PCR-based method did not retrieve the genera found with the culture methods conducted on midgut bacteria of two field-collected *Anopheles* species (*An*. *gambiae* and *An*. *funestus*) from Western Kenya. Herein, the discordances were observed at each taxonomic level, even at the phylum level, and particularly concerning Gram-negative and Gram-positive phyla, containing bacteria differing in their peptidoglycan layer structure [58]. This peptidoglycan layer allows Gram-positive bacteria to be more stable to the lysis buffer reaction than Gram-negative bacteria [59]. This is particularly so for *Actinobacteria* because of an unusual cell envelope composition, characterized by the presence of a waxy cell envelope containing mycolic acids [60]. Despite the cell wall lysis method used enhanced Gram-positive lysis, we hypothesize that partial cell lysis before DNA extraction introduced a bias in favor of Gram-negative bacteria leading to an under-representation of Gram-positive phyla in the DNA-based approach. Finally, each approach of determining bacterial diversity presented potential biases, the non-cultivability of certain bacteria being the more obvious, but potential biases associated with molecular methods must also be considered. Previous results obtained by Next Generation Sequencing (NGS) and fingerprinting by TTGE show good correlation for the detection of majority OTUs in complex communities [37,61]. In the context of this descriptive study of the diversity of *Anopheles* microbiota in Vietnam, the detection of major populations and their variation is deemed a sufficient first step, but subsequent in-depth NGS should be done on the subset of parasite-associated and parasite-free mosquitoes.

For malaria control, many strategies have been implemented with varying success. The blocking of *Plasmodium* transmission, based on bacterial genetic changes to deliver molecules or as a paratransgenic approach, is a relatively new concept and strategy [62]. Several studies have been conducted on the characterization of the bacterial flora in the midgut of *Anopheles* to determine the bacterial candidate(s) for effectively blocking malaria transmission [19,63]. Several methods have been applied, including culture-dependent detection of bacteria based on colony isolation on solid medium, sometimes following enrichment in liquid medium and culture-independent methods based directly on molecular techniques. Some studies have relied on either culture-dependent [14,20] or culture-independent techniques [23,35], while others, as in our study, have favored the power of combining these two methods for evaluating the bacterial diversity *Anopheles* midguts (abdominal cavity) [15,22]. The molecular approach showed more sensitivity than plate culturing alone with bacteria detected in half of the samples (26 of 52 specimens), while only a quarter of the samples (13/52) were positive by culture methods. However, the sizes of the sequences obtained from TTGE products and some NGS were short (∼200 bp) resulting in the lack of genetic information and thus impeding an accurate affiliation to the species taxonomic level [37]. The use of NGS could improve the length of DNA fragments to around 500 bp [64]. Culture is the classical approach to study bacterial communities and allows access to the complete 16S rRNA gene sequences from culture bacterial isolates and an accurate affiliation to the lowest taxonomic level [65]. It also provides advantages of using living bacteria for further functional investigations and experiments. Conversely, the culture-independent techniques allow the detection of bacteria that are difficult to develop on normal culture mediums or those requiring specific culture conditions for propagation such as anaerobic bacteria [66,67]. The combination of both methods thus allows the detection of a larger panel of bacteria diversity which is the first step in the investigation of those microbiota that might possibly be involved in interfering or preventing pathogen development in the mosquito (e.g., *Plasmodium* sporogonic development in the *Anopheles* midgut) [38,68]. A better knowledge of the full array of bacteria and other microorganisms that coexist in mosquitoes is the first step to discovering the potential of new and novel methods of disease control.

### Conclusion

Based on culture-dependent and culture-independent methods, we found *Anopheles* specimens from Dak Nong, Vietnam contained a great diversity of bacteria in their abdomen, including bacteria species previously implicated in influencing the development of malaria parasites in mosquitoes. As various microbiota might have significant ability for suppressing or preventing pathogen development in *Anopheles* mosquitoes and thus parasite transmission, the study of the midgut microbiota of *Anopheles* vectors must be promoted. This has become an even more pressing issue, as other forms of disease and vector control are under constant pressure and the need for new tools an urgent mandate. For example, the use of insecticides for vector control has been compromised because of high levels of resistance in numerous vector populations, or the development and spread of drug resistant parasites. This first study reporting the biodiversity of microbiota of *Anopheles* in Vietnam should lead to further study to better understand the disease-modulating role of specific bacteria isolated from wild mosquito populations for developing new approaches in controlling *Anopheles* vectors and malaria transmission in Vietnam.

## Supporting Information

S1 Fig Rarefaction curve showing the number of OTUs in relation to the number of sequences. (TIFF)

S1 Dataset Minimal dataset with the raw data. (XLSX)
