# Supplementary figures and images for: Bacterial Diversity Associated with Wild Caught Anopheles Mosquitoes from Dak Nong Province, Vietnam Using Culture and DNA Fingerprint
Source: PLoS One. 2015 Mar 6;10(3):e0118634. doi: 10.1371/journal.pone.0118634 (PMC4352016; doi:10.1371/journal.pone.0118634)

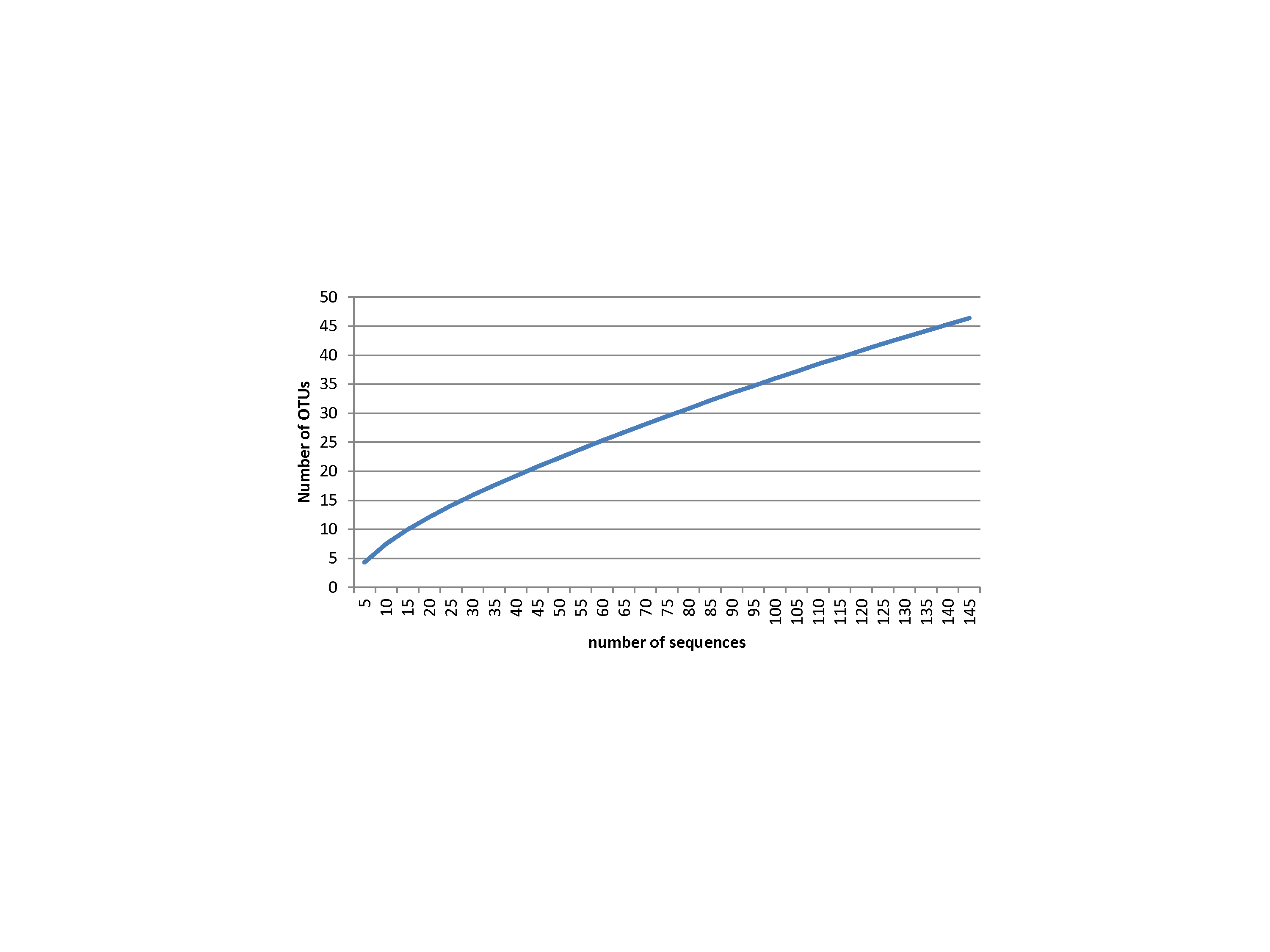

Supplement: S1 Fig — (TIFF) [file pone.0118634.s001.tiff]
